# An Open‐Label, Single‐Arm, Phase II Trial of Sintilimab Plus Anlotinib for Metastatic Non‐Small Cell Lung Cancer After First‐Line PD‐(L)1 Inhibitor

**DOI:** 10.1002/cam4.71191

**Published:** 2025-09-04

**Authors:** Xun Shi, Chen Lin, Lishu Lou, Qiong He, Guangyuan Lou, Wei Hong, Lan Shao, Jun Zhao, Cuiping Gu, Xinmin Yu, Ying Jin

**Affiliations:** ^1^ Zhejiang Cancer Hospital, Hangzhou Institute of Medicine (HIM), Chinese Academy of Sciences Hangzhou China

**Keywords:** anti‐angiogenic therapy, combination, immunotherapy, non‐small‐cell lung cancer, previously treated

## Abstract

**Background:**

Although immune checkpoint inhibitors (ICIs) have markedly improved first‐line management of non‐small cell lung cancer (NSCLC), many tumors eventually escape control after anti‐PD‐(L)1 therapy, leaving a clear therapeutic gap. Preclinical studies and preliminary clinical data suggest that coupling ICIs with anti‐angiogenesis therapy can yield complementary antitumor effects. Consequently, we launched this investigation to evaluate the therapeutic benefit and tolerability of sintilimab, a PD‐(L)1–blocking monoclonal antibody, together with the oral multi‐target anti‐angiogenesis agent anlotinib in metastatic NSCLC individuals experiencing progression after first‐line PD‐(L)1 inhibition.

**Methods:**

At Zhejiang Cancer Hospital, we conducted a phase II trial, single‐arm, open‐label investigation (registration No. NCT04691388). Patients were adults diagnosed with metastatic NSCLC whose disease had advanced after PD‐(L)1 blockade; they received sintilimab plus anlotinib on a 21‐day cycle. Investigator‐assessed objective response rate (ORR) served as the principal efficacy endpoint.

**Results:**

Between March 2021 and January 2024, twenty‐nine individuals were recruited (median age 63, range 45–74, 96.6% male). Tumor assessment identified five partial responses (PR) (17.2%), nineteen cases of stable disease (SD) (65.5%) and three progressions (10.3%), yielding an ORR of 17.2% and a disease‐control rate (DCR) of 82.8%. The cohort's median progression‐free survival (PFS) measured 5.0 months (90% CI, 4.2–7.3), and 41.1% of participants remained progression‐free at six months. Overall survival reached a median of 15.1 months (90% CI, 8.6–not yet reached), with an 18‐month survival proportion of 44.8%. Grade ≥ 3 treatment‐related toxicity was dominated by hypertension, occurring in 10.3% of participants; no patient discontinued therapy or died because of drug‐related events.

**Conclusion:**

Sintilimab combined with anlotinib exhibited favorable antitumor activity and tolerable toxicity in post‐anti‐PD‐(L)1 therapy of metastatic NSCLC patients, supporting further randomized controlled trials.

**Trial Registration:**

ClinicalTrials.gov: NCT04691388. Registered December 31, 2020

**Abbreviations**
AEs: adverse events
CTCAE: common terminology criteria for adverse events
DCR: disease‐control rate
ECOG: Eastern Cooperative Oncology Group
ICIs: immune‐checkpoint inhibitors
NMPA: National Medical Products Administration
NSCLC: non‐small cell lung cancer
ORR: overall response rate
OS: overall survival
PD: progressive disease
PD‐1: programmed cell‐death protein 1
PD‐L1: programmed cell‐death ligand 1
PFS: progression‐free survival
PR: partial response
RECIST: response evaluation criteria in solid tumors
SD: stable disease
TME: tumor micro‐environment
TRAEs: treatment‐related adverse events

## 1 Introduction

GLOBOCAN 2022 still identifies pulmonary carcinoma as the leading cause contributor to global cancer mortality, accounting for nearly an estimated 1.8 million lives lost—approximately 18.7% of all oncology‐related deaths in 2022 [1]. These staggering mortality figures underscore the urgency for effective therapeutic strategies, particularly in the driver‐negative population where targeted therapies are inapplicable. In advanced non‐small‐cell lung cancer (NSCLC) cases—either metastatic or locally progressive—without targetable driver alterations, PD‐(L)1 axis inhibition has emerged as the preferred first‐line therapy, offering substantial survival advantages compared to historical standards [2, 3, 4, 5, 6, 7, 8].

Although immune checkpoint–targeted approaches have markedly extended overall survival (OS) for individuals with late‐stage NSCLC, many patients ultimately fail to derive lasting benefit from these treatments [9]. Once tumors progress after PD‐(L)1 inhibition, guideline‐endorsed management reverts to cytotoxic chemotherapy, with docetaxel representing the principal option [10]. However, historical data have shown that these drugs provide limited survival benefits after treatment and are associated with toxic reactions [11, 12], highlighting a significant unmet therapeutic need in patients occurring immune resistance. Subgroup analyses from multiple randomized clinical trials revealed that9% to 48% of patients persisted with anti‐PD‐(L)1 therapy for 4 to 6 weeks following disease progression after first‐line treatment. Within this cohort,13% to 33% of patients achieved at least a 30% decrease in the sum of target‐lesion diameters relative to measurements taken after disease progression [13, 14, 15, 16, 17]. Moreover, individuals who re‐initiated PD‐(L)1 blockade experienced longer progression‐free survival (PFS) and OS than those who permanently discontinued immunotherapy following first‐line failure [18].

The curative effect of immunotherapy is closely associated with the immune infiltration status of the tumor microenvironment (TME), while anti‐angiogenic drug therapy not only participates in the remodeling of abnormal blood vessels in the TME and modulates the infiltration of tumor‐associated immune cells, but also reverses the immunosuppressive status of TME [19]. The combination of the two treatments has a potential synergistic mechanism of action, offering promising prospects for their combined application in antitumor therapy. The phase III IMpower150 study was the first to pair an anti‐angiogenic agent with immunotherapy. In that study, adding bevacizumab to atezolizumab improved outcomes, extending the median PFS to 8.3 months, whereas it was 6.8 months for the control cohort (HR = 0.62) and raising OS to 19.2 months, compared with 14.7 months among controls (HR = 0.78) [20]. Similarly, the ORIENT‐31 trial tested a four‐agent regimen consisting of sintilimab, bevacizumab, and platinum doublet chemotherapy; relative to chemotherapy by itself, the combination increased median PFS to 7.2 months versus 4.3 months and achieved an objective response rate (ORR) of 48% [21].

Sintilimab is a recombinant, fully human IgG4 antibody that targets PD‐1 with higher binding affinity than either pembrolizumab or nivolumab, according to pre‐clinical assays. In a double‐blind phase III trial, first‐line sintilimab plus platinum‐based chemotherapy significantly extends PFS and OS in advanced NSCLC lacking EGFR or ALK driver mutations. Notably, this combination therapy maintains a favorable safety profile [3, 8]. Anlotinib blocks tumor angiogenesis and proliferation by simultaneously inhibiting VEGFR‐1/−2/−3 and multiple other tyrosine‐kinase receptors [22]. Participants were randomized into two groups: one receiving anlotinib (*n* = 296) and the other a placebo (*n* = 143). Anlotinib produced a marked PFS advantage, with median PFS rising to 5.4 months versus 1.4 months for placebo (*p* < 0.0001). Median OS was likewise prolonged, reaching 9.6 months compared with 6.3 months in the control arm (*p* = 0.0018) [23]. Drawing on these findings, the Chinese National Medical Products Administration (NMPA) granted approval to anlotinib in May 2018 for use as a third‐line therapy in metastatic NSCLC after failure of at least two systemic chemotherapy regimens. More recently, first‐line administration of sintilimab plus anlotinib in advanced NSCLC yielded an ORR of 72.7% and a DCR of 100%, indicating a promising immunotherapeutic strategy [24]. However, the effect of this regimen on previously PD‐(L)1 treated patients is vague. Building on this approval, we designed a prospective trial to explore the efficacy and tolerability of combining sintilimab with anlotinib in patients whose NSCLC advanced after first‐line PD‐(L)1 antibody therapy.

## 2 Materials & Methods

### 2.1 Study Design and Participants

This Phase II clinical trial adopted a prospective, single‐center, single‐arm design and was conducted at Zhejiang Cancer Hospital located in Hangzhou, China. The study protocol received institutional review board approval before the enrollment of the first participant.

This research was designed to assess the therapeutic efficacy and tolerability of anlotinib–sintilimab regimen in adult patients diagnosed with advanced‐stage NSCLC that had progressed following initial PD‐1/PD‐L1 blockade. Ethical approval for the study was granted by the Zhejiang Cancer Hospital review board, and all participants provided signed consent forms prior to enrollment. Eligible subjects were 18 to 75 years old, carried stage IV disease per the eighth AJCC edition, and had radiological progression under RECIST v1.1 following initial PD‐1/PD‐L1 blockade. Further criteria included an Eastern Cooperative Oncology Group (ECOG) performance status of 0 to 1, the presence of at least one RECIST‐v1.1–measurable lesion, anticipated survival of ≥ 3 months, and no activating EGFR, ALK, or ROS1 mutations.

Exclusion criteria encompassed: (i) histological evidence of small cell lung cancer—even if admixed with NSCLC; (ii) previous exposure to immune‐modulating antibodies other than PD‐(L)1 blockade, such as anti‐CTLA‐4 and other similar agents; (iii) prior use of anlotinib or other VEGFR‐targeted tyrosine‐kinase inhibitors such as sorafenib, sunitinib, famitinib, apatinib, or regorafenib; (iv) CT/MRI demonstrating a tumor ≤ 5 mm from a major vessel, invasion of a central vessel, or marked cavitation/necrosis within the lung lesion; and (v) unacceptable toxicity experienced during earlier PD‐(L)1 inhibitor.

### 2.2 Procedures

All patients received sintilimab and anlotinib on a three‐week cycle. Participants were administered intravenous sintilimab 200 mg on the first day of every 21‐day treatment cycle, together with oral anlotinib 12 mg once daily for 14 days, followed by a 7‐day break. Participants who achieved clinical benefit—as determined per RECIST 1.1 criteria by imaging‐confirmed complete response (CR), stable disease (SD), or partial response (PR)—remained on study treatment until meeting protocol‐defined discontinuation criteria, which included: (1) radiological progression confirmed by central imaging review; (2) immune‐related adverse events (irAEs) of grade 3 or above classified according to CTCAE v5.0; (3) investigator‐determined loss of clinical benefit; (4) completion of 35‐cycle exposure (24‐month therapeutic window). Concurrent antitumor interventions were strictly prohibited during protocol‐specified evaluation periods.

Sintilimab dosing was fixed; no reductions were permitted. Conversely, anlotinib could be tapered to 10 mg or 8 mg once daily—or ceased entirely—at the investigator's discretion should treatment‐related toxicity arise.

Tumor assessments were conducted every two cycles, and patients achieving CR, PR, or SD were re‐evaluated 6 weeks after the initial assessment. Patients were reviewed every 8 weeks via imaging, clinical visits, or telephone contacts to document tumor response, adverse effects, and survival, with follow‐up continuing until death or loss to follow‐up.

### 2.3 Outcomes

The chief efficacy endpoint was the ORR as judged by investigators, defined as the percentage of participants achieving either complete or partial tumor regression under RECIST 1.1. Key secondary outcomes comprised PFS (RECIST v1.1), OS, DCR, and safety. PFS constituted the primary efficacy metric, operationally measured from initial therapeutic intervention until the earliest occurrence of radiologically confirmed disease advancement (per RECIST 1.1) or all‐cause mortality, whichever manifested first. OS refers to the duration spanning from treatment initiation date until death from any cause occurred. DCR represents the percentage of subjects who achieved CR, PR, or SD. Adverse events, both treatment‐associated and immune‐related, were tracked for frequency, type, and intensity. Each event was recorded, with severity classified using the National Cancer Institute Common Terminology Criteria for Adverse Events (CTCAE v5.0).

### 2.4 Statistical Analysis

This phase II clinical trial methodology employed Simon's optimal two‐stage design, incorporating predefined statistical parameters for ORR thresholds aligned with RECIST 1.1 criteria. Referring to historical data, the ORR of second‐line single‐agent chemotherapy for driver gene‐negative NSCLC was 8% [11], and the ORR of the alternative hypothesis was set at 25%. Employing Simon's two‐stage optimal methodological design with prespecified parameters (one‐sided *α* = 0.05, *β* = 0.2), this prospective study successfully enrolled a cohort of 26 eligible participants. A Simon two‐stage design was adopted. Stage 1 enrolled 20 patients; if no more than two objective responses (complete or partial) were recorded, the study would halt. Otherwise, recruitment advanced to stage 2. The null hypothesis would be discarded when the overall number of objective responses surpassed four. Allowing for an anticipated 10% dropout, a total of 29 participants were planned.

The full analysis population included every participant who had taken at least one dose of study medication, excluding major protocol violators. The safety set included every treated subject who had post‐baseline safety data. Statistical analyses were conducted with R 4.3.1. For normally distributed data, results are shown as mean ± standard deviation; skewed variables are summarized by median and range, whereas categorical data are reported as counts and percentages. Response rates with associated precision estimates (90% confidence intervals) were calculated via the exact binomial interval procedure described by Clopper and Pearson. FS, OS, and their 90% confidence intervals were estimated using Kaplan–Meier methodology. Safety outcomes were summarized primarily with descriptive statistics. Treatment‐related adverse events (TRAEs) were systematically classified according to CTCAE 5.0 grading criteria, with comprehensive categorization by toxicity severity and organ system involvement, sorted by descending frequency.

## 3 Results

### 3.1 Patient Characteristics

Figure S1 outlines the enrollment pathway. Between March 2021 and January 2024, 30 individuals entered the trial and received ≥ one administration of the investigational therapy; one participant was subsequently excluded because an off‐protocol surgical procedure was performed. The remaining 29 subjects formed both the efficacy and safety populations. At the data cut‐off of 17 October 2024, treatment continued in two patients, whereas 27 had stopped therapy—22 owing to radiographic progression, two because of study withdrawal, two after death, and one following withdrawal of consent (Figure S1).

Table 1 presents a concise overview of the participants' baseline demographics and disease characteristics. The median age of the cohort was 63 years (range 45–74). Most patients were men (28/29, 96.6%) and had a smoking history (23/29, 79.3%). All enrolled patients exhibited an ECOG performance status of 1. Sixteen (55.2%) patients had a histological type of adenocarcinoma, and the remaining patients were squamous cell carcinoma. Of the 29 patients, 5 (17.2%) had bone metastasis, 9 (31.0%) had hepatic metastases, and 3 (10.3%) had brain metastases.

**TABLE 1:** Baseline characteristics.

	Overall (*N* = 29)
*Age*	
Median	63
Range	45–74
*Gender*	
Female	1 (3.4%)
Male	28 (96.6%)
*Smoking history*	
No	6 (20.7%)
Yes	23 (79.3%)
*ECOG PS*	
1	29 (100.0%)
*Histological classification*	
Adenocarcinoma	16 (55.2%)
Squamous cell carcinoma	13 (44.8%)
*Bone metastasis*	
No	24 (82.8%)
Yes	5 (17.2%)
*Hepatic metastases*	
No	20 (69.0%)
Yes	9 (31.0%)
*Brain metastases*	
No	26 (89.7%)
Yes	3 (10.3%)

### 3.2 Efficacy

**FIGURE 1:**
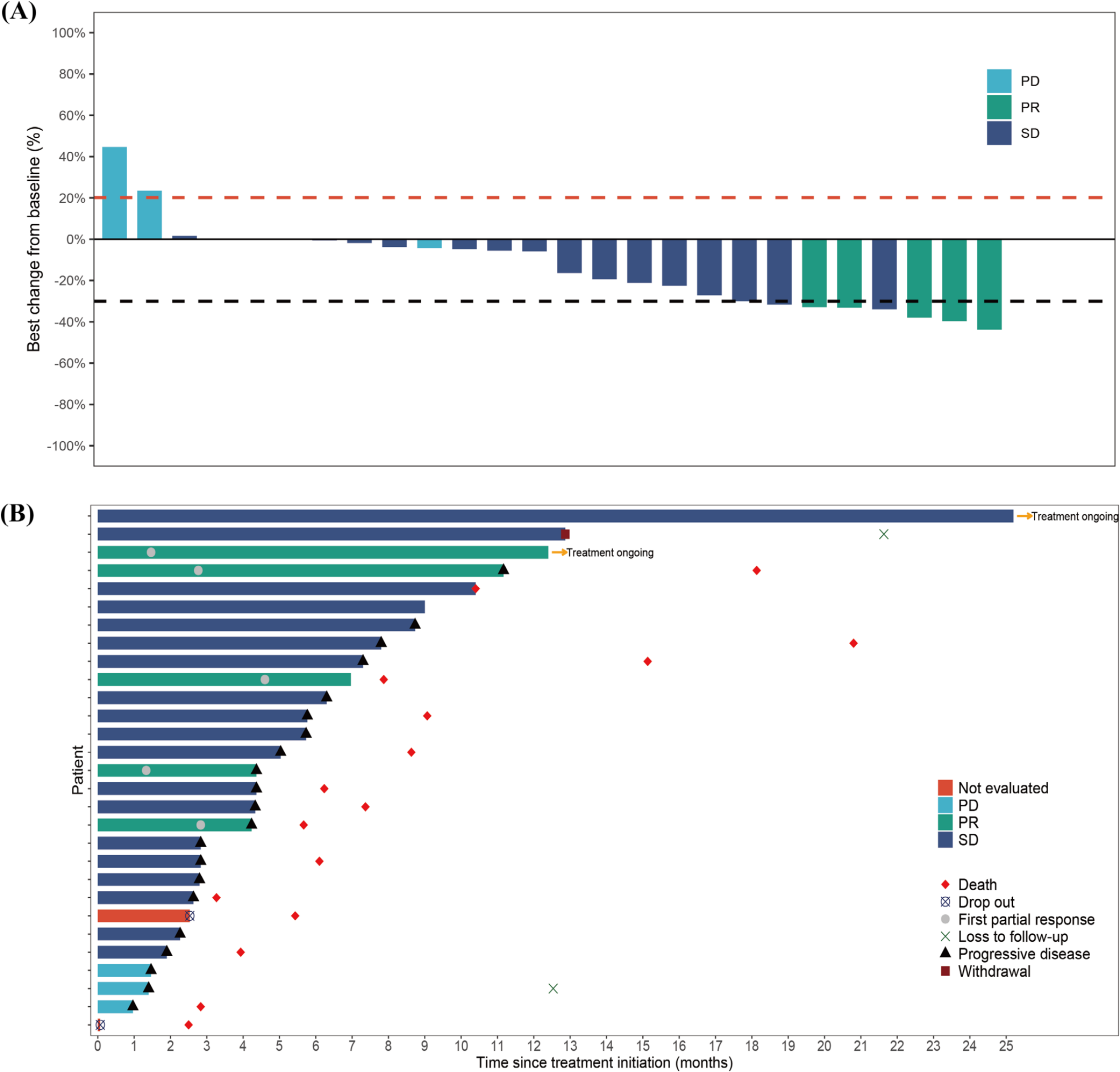
(A) Best percentage changes from baseline in target lesion size in the efficacy analysis set. (B) Treatment response and duration of response. CR, complete response; PD, disease progression; PR, partial response; SD, stable disease.

Of the 22 patients enrolled in the first stage, 3 (13.6%) achieved the PR, exceeding the prespecified threshold of 2, and thus the trial proceeded to the second stage. Among the 29 patients, 27 (93.1%) were evaluable for tumor response, with 5 (17.2%) PR, 19 (65.5%) SD, and 3 (10.3%) progressive disease (PD) (Table 2). The ORR was 17.2% (90% CI: 7.0%–32.9%) and the DCR was 82.8% (90% CI: 67.1%–93.0%) in overall patients. Figure 1A displays percentage variations from baseline in the aggregate diameter of target lesions (RECIST v1.1); tumor reduction was recorded in 20 of 29 patients (69.0%). Individual best overall responses and response durations are depicted in Figure 1B.

**TABLE 2:** Tumor response.

Overall response	*N* = 29
PR	5 (17.2%)
SD	19 (65.5%)
PD	3 (10.3%)
NE	2 (6.9%)
ORR (90% CI)	17.2% (7.0%–32.9%)
DCR (90% CI)	82.8% (67.1%–93.0%)

Abbreviations: CI, confidence interval; DCR, disease control rate; NE, not evaluated; ORR, objective response rate; PD, progressive disease; PR, partial response; SD, stable disease.

**FIGURE 2:**
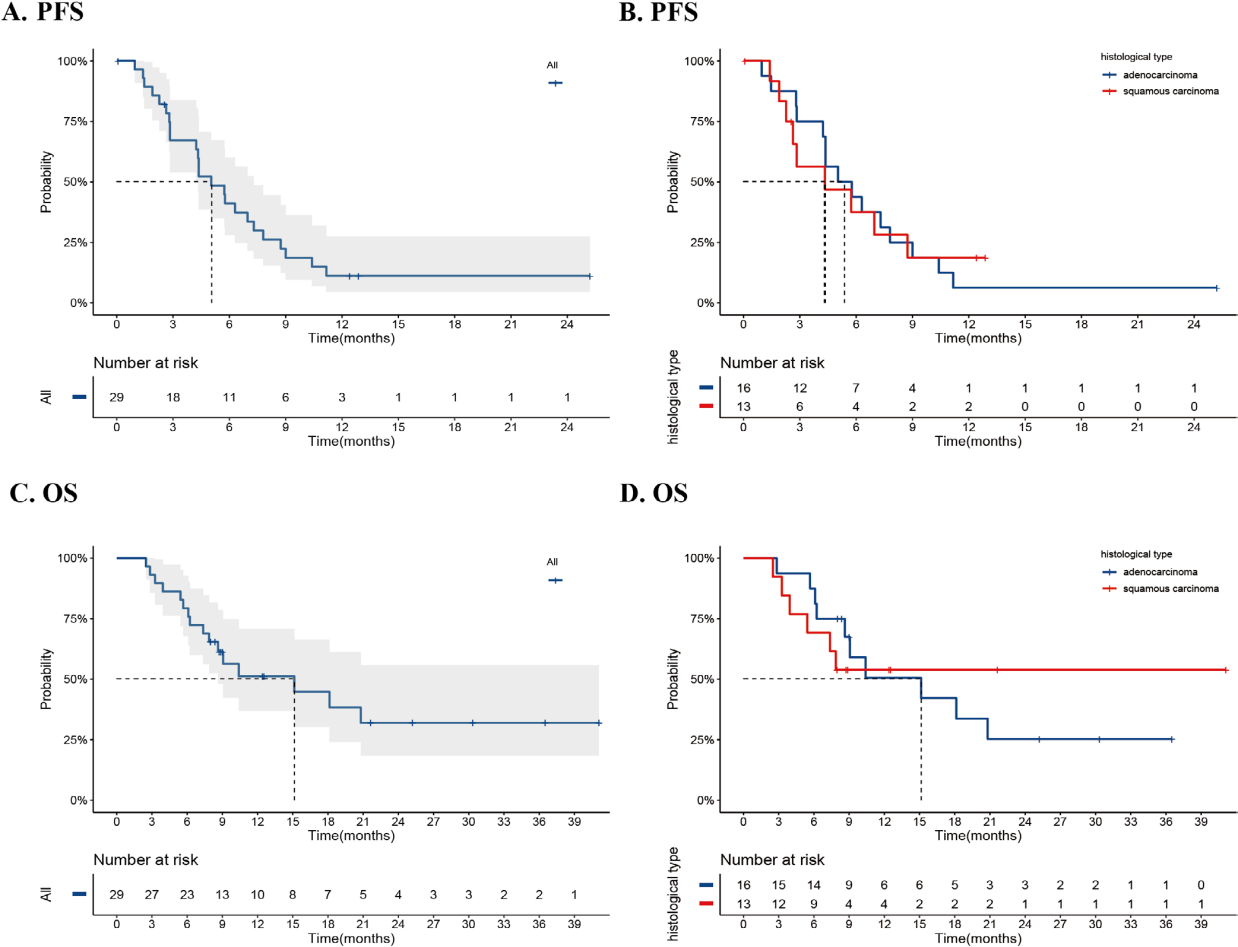
Analysis of PFS. (A) All patients. (B) Nonsquamous versus squamous; Analysis of OS. (C) All patients. (D) Nonsquamous versus squamous. OS, overall survival; PFS, progression‐free survival.

At the data‐cutoff date, patients had been followed for a median of 21.6 months (95% CI: 9.0–not reached [NR]). Disease progression had occurred in 22 participants, and 16 deaths had been recorded. Figure 2 illustrates Kaplan–Meier survival estimates for both PFS and OS, including event timelines and confidence intervals. The median PFS was 5.0 months (90% CI, 4.2–7.3), corresponding to a PFS rate of 41.1% at 6 months and 11.2% at 12 months (Figure 2A). Median OS reached 15.1 months (90% CI, 8.6–NR), with 18‐ and 24‐month survival rates of 44.8% and 32.0%, respectively (Figure 2C). Subgroup analysis showed a higher 12‐month PFS rate in squamous tumors than in adenocarcinoma (18.8% vs. 6.3%). Median PFS was 4.3 months for squamous carcinoma and 5.4 months for adenocarcinoma (Figure 2B). Median OS was NR in the squamous subgroup, whereas it was 15.1 months in adenocarcinoma (Figure 2D).

**FIGURE 3:**
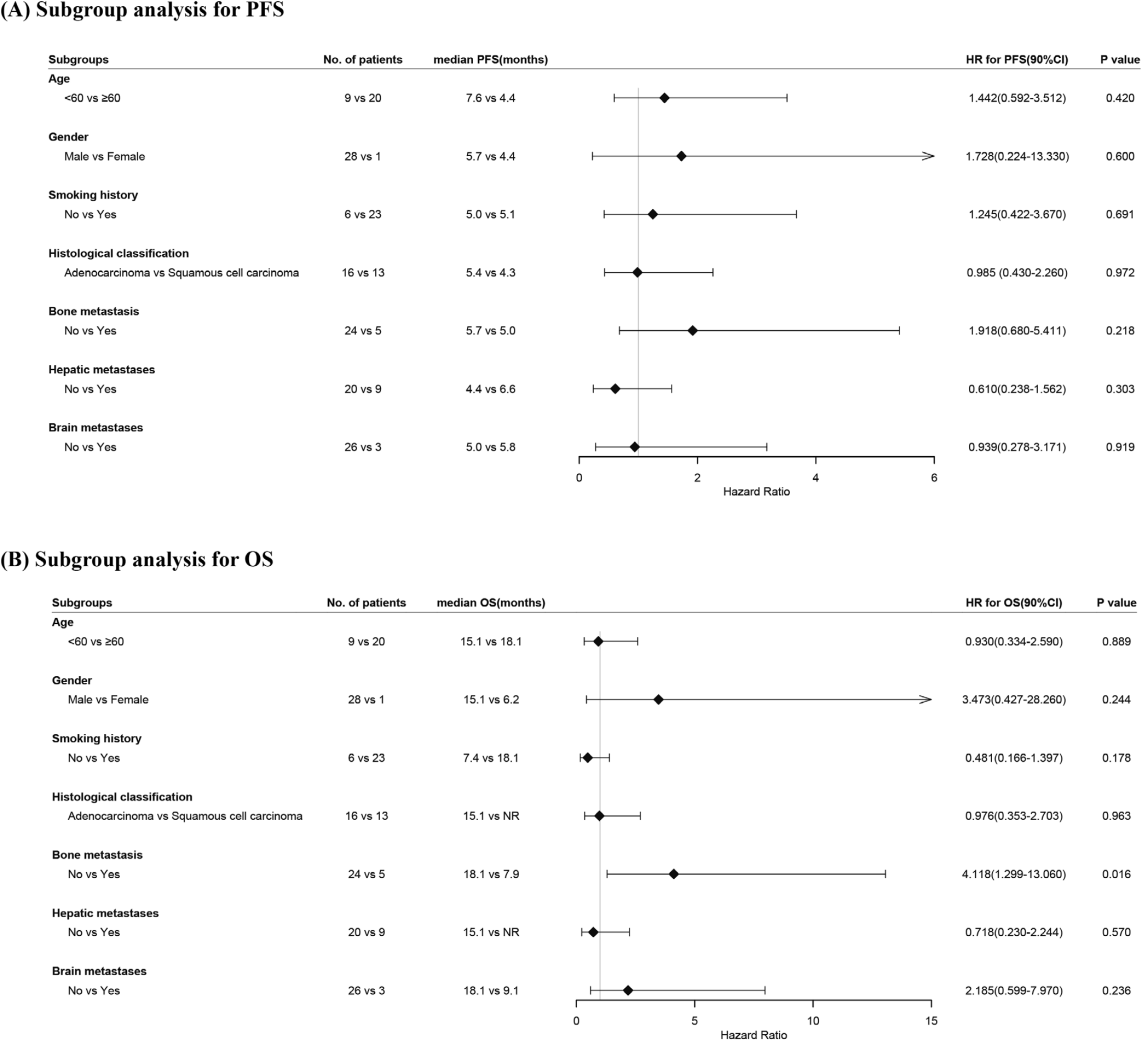
Subgroup analysis of (A) PFS and (B) OS. CI, confidence interval; HR, hazard ratio; OS, overall survival; PFS, progression‐free survival.

Subgroup analysis demonstrated that bone metastasis status (presence vs. absence) served as a significant prognostic factor for OS. Patients with bone metastases demonstrated a significantly shorter median OS (7.9 vs. 18.1 months; HR = 4.118, 90% CI: 1.299–13.060, *p* = 0.016) compared to those without metastatic bone involvement (Figure 3B). When squamous and non‐squamous histologies were compared, no meaningful differences emerged: median PFS values were 5.4 and 4.3 months (HR = 0.985; 90% CI 0.430–2.260; *p* = 0.972), and median OS was 15.1 months versus (HR = 0.976; 90% CI 0.353–2.703; *p* = 0.963) after anti‐angiogenic plus immune therapy in the post‐PD‐(L)1 setting (Figure 3).

### 3.3 Safety

Any grade TRAEs occurred in 25 of 29 patients (86.2%). Most common TRAEs (frequency of ≥ 10%) of any grade were proteinuria (14/29, 48.3%), hypertension (11/29, 37.9%), hypothyroidism (7/29, 24.1%), hand‐foot syndrome (6/29, 20.7%), anemia (4/29, 13.8%), dental ulcer (4/29, 13.8%), hyponatremia (3/29, 10.3%), hematuria (3/29, 10.3%), increased lactate dehydrogenase (3/29, 10.3%), and pneumonia (3/29, 10.3%) (Table 3). TRAEs of grade ≥ 3 occurred in 9 of 29 patients (31.0%), most commonly hypertension (3/29, 10.3%). No participant discontinued the investigational regimen because of drug‐related toxicity, and no deaths attributable to treatment occurred.

**TABLE 3:** TRAEs (frequency of ≥ 5%).

Adverse events	Grade 1, *n* (%)	Grade 2, *n* (%)	Grade 3, *n* (%)	Any grade, *n* (%)
Proteinuria	10 (34.5)	4 (13.8)	0 (0)	14 (48.3)
Hypertension	3 (10.3)	5 (17.2)	3 (10.3)	11 (37.9)
Hypothyroidism	3 (10.3)	4 (13.8)	0 (0)	7 (24.1)
Hand‐foot syndrome	1 (3.4)	4 (13.8)	1 (3.4)	6 (20.7)
Anemia	4 (13.8)	0 (0)	0 (0)	4 (13.8)
Dental ulcer	2 (6.9)	2 (6.9)	0 (0)	4 (13.8)
Hyponatremia	1 (3.4)	1 (3.4)	1 (3.4)	3 (10.3)
Haematuria	3 (10.3)	0 (0)	0 (0)	3 (10.3)
Increased lactate dehydrogenase	3 (10.3)	0 (0)	0 (0)	3 (10.3)
Pneumonia	2 (6.9)	1 (3.4)	0 (0)	3 (10.3)
Increased glutamic oxalacetic transaminase	1 (3.4)	1 (3.4)	0 (0)	2 (6.9)
Whole‐body ache	0 (0)	2 (6.9)	0 (0)	2 (6.9)
Increased γ‐glutaminase transferase	1 (3.4)	1 (3.4)	0 (0)	2 (6.9)
Increased direct bilirubin	2 (6.9)	0 (0)	0 (0)	2 (6.9)
Increased Creatine kinase isoenzyme	2 (6.9)	0 (0)	0 (0)	2 (6.9)
Dull sore throat	1 (3.4)	1 (3.4)	0 (0)	2 (6.9)
Urinary tract infection	2 (6.9)	0 (0)	0 (0)	2 (6.9)

## 4 Discussion

In summary, this trial demonstrates that a chemotherapy‐sparing strategy consisting of sintilimab and anlotinib is both active and tolerable for metastatic NSCLC progressing on prior PD‐(L)1 blockade. The research demonstrated encouraging treatment outcomes, with ORR of 17.2%; median PFS reached 5.0 months, and OS extended to 15.1 months. The sintilimab‐anlotinib regimen demonstrated favorable tolerability with a relatively low frequency of grade ≥ 3 TRAEs, particularly exhibiting reduced hematologic toxicity profiles relative to chemotherapy‐based approaches. We all know that the most significant drawback of chemotherapy lies in its frequent hematologic toxicity. Our study employed a chemotherapy‐free regimen, demonstrating only a 13% incidence of anemia among enrolled patients with no other observable hematologic toxicity events. Thus, sintilimab plus anlotinib represented an encouraging chemo‐free option for advanced NSCLC who have been previously treated with anti‐PD‐(L)1 immunotherapy.

Over the past few years, multiple trials have assessed anti‐angiogenic agents paired with immunotherapy in advanced NSCLC that has become refractory to immune checkpoint blockade, seeking to determine whether these regimens outperform docetaxel. Regrettably, studies such as SAPPHIRE [25], CONTACT‐01 [26], LEAP‐008 [27] and SAFFRON‐301 [28] have generally failed to demonstrate statistically significant superiority over docetaxel in terms of efficacy. A significant contributing factor to this outcome may be the relatively pronounced adverse effects associated with drugs like lenvatinib, sitravitinib, and cabozantinib. In the SAFFRON‐301 study [28], patients in the investigational arm had a greater incidence of grade ≥ 3, serious, and discontinuation‐related TRAEs compared to those treated with docetaxel. In addition to several antivascular drugs mentioned above, anlotinib displays a good therapeutic effect on NSCLC with a relatively mild toxicity and is now recommended in China for use from the third‐line setting onward. In the ALTER 0303 trial [23], anlotinib prolonged OS by 3.3 months (HR 0.68) and extended PFS by 4.0 months (HR 0.25) advanced NSCLC patients previously treated with two or more systemic regimens. The earliest investigation pairing a PD‐(L)1 inhibitor with a broad‐spectrum anti‐angiogenic TKI in the first‐line setting was a phase I trial evaluating sintilimab plus anlotinib in treatment‐naïve, driver‐mutation–negative stage IIIB–IV NSCLC. A newly published phase II randomized controlled study initiated by Prof. Han Baohui demonstrated that initial therapy with sintilimab combined with anlotinib achieved superior ORR and PFS versus standard platinum chemotherapy in metastatic NSCLC, while also offering a more favorable safety profile [29]. Moreover, the combination of anlotinib with benmelstobart produced encouraging tumor responses in advanced NSCLC lacking EGFR or ALK alterations, with generally manageable toxicities [30]. Numerous studies have corroborated the efficacy and tolerability of anlotinib in patients who had previously received immune checkpoint blockade [31, 32].

Contrary to previous studies [25, 26, 27, 28], this study reveals comparable therapeutic benefits of anlotinib in both squamous and non‐squamous NSCLC subtypes, potentially indicating mechanistic distinctions from other antiangiogenic agents. The observed efficacy divergence may stem from anlotinib's unique dual‐pathway activity: sustained angiogenesis modulation through vascular endothelial stabilization and concurrent immunomodulatory effects via PD‐L1+ macrophage regulation. Notably, the study pioneers bone metastasis stratification analysis, demonstrating significantly inferior progression‐free survival in osseous metastatic subgroups (median PFS 3.2 vs. 5.8 months; HR 1.92, 95% CI 1.13–3.28, *p* = 0.016)—a critical prognostic determinant previously unaddressed in angiogenesis inhibitor trials. These findings necessitate meticulous clinical evaluation when administering anlotinib to patients with skeletal metastases.

Notably different from previous trials, this study exclusively enrolled metastatic NSCLC patients with ECOG performance status 1, further highlighting that superior therapeutic efficacy was achieved in a population with inferior baseline characteristics. These findings suggest that the sintilimab‐anlotinib combination may represent an effective therapeutic strategy for immunotherapy‐resistant cases. Significantly, our study demonstrated a superior median OS of 15.1 months, exceeding results from all existing comparable clinical trials: the CONTACT‐01 trial [26] reported 10.9 months for atezolizumab plus cabozantinib in immunotherapy‐resistant metastatic NSCLC, the SAFFRON‐301 study [28] achieved 11.7 months with the tislelizumab‐sitravatinib combination, while the LEAP‐008 trial [27] observed 11.3 months for pembrolizumab‐levatinib therapy. Superior to these studies, our trial prospectively confirmed the efficacy of the combination therapy in previously PD‐(L)1 treated patients, providing a clue to delay immuno‐resistance as a second‐line therapy for driver‐gene negative NSCLC.

The principal weakness of this investigation is its single‐arm structure, which obliges comparison with historical control cohorts and may consequently introduce selection bias. Additionally, the lack of mandatory PD‐L1 testing at enrollment resulted in unavailable biomarker data for 72.4% (21/29) of patients, precluding meaningful analysis of its potential relationship with treatment outcomes. Randomized controlled trials should be designed to further investigate and validate the preliminary results of this study and to explore the underlying mechanisms. Although the median OS was achieved at the time of data cutoff, the upper limit of the confidence interval for OS has not been reached, so long‐term follow‐up is requisite to assess the ultimate survival outcomes. Moreover, only Chinese participants were recruited in the study, and it is plausible that treatment efficacy and tolerability differ across other ethnic groups; this possibility should be explored in future multinational trials.

## 5 Conclusion

In summary, our findings suggest that the combination of sintilimab and anlotinib delivers meaningful clinical activity with acceptable toxicity in driver‐negative advanced NSCLC progressing after first‐line PD‐1 blockade, and thus warrants consideration as an additional therapeutic choice.

## Author Contributions

**Xun Shi:** writing – original draft, project administration, data curation. **Chen Lin:** writing – original draft, project administration, data curation. **Lishu Lou:** writing – review and editing, formal analysis, visualization. **Qiong He:** writing – review and editing, project administration, data curation. **Guangyuan Lou:** data curation, project administration, writing – review and editing. **Wei Hong:** writing – review and editing, project administration, data curation. **Lan Shao:** data curation, project administration, writing – review and editing. **Jun Zhao:** writing – review and editing, project administration, data curation. **Cuiping Gu:** data curation, project administration, writing – review and editing. **Xinmin Yu:** project administration, writing – review and editing, conceptualization, methodology, data curation. **Ying Jin:** conceptualization, methodology, project administration, writing – review and editing, data curation.

## Ethics Statement

The study protocol was approved by the ethics committee of Zhejiang Cancer Hospital, and was carried out in accordance with the Helsinki Declaration and ICH Good Clinical Practice guidelines. All participants signed written informed consent before any study‐specific procedures were undertaken.

## Consent

The authors have nothing to report.

## Conflicts of Interest

The authors declare no conflicts of interest.

## Supporting information


**Figure S1:** cam471191‐sup‐0001‐Figure_S1.docx.

## Data Availability

The raw dataset analyzed and utilized in this study can be accessed through formal request to the principal investigator, contingent upon providing reasonable justification.
