# Supplementary figures and images for: An Open‐Label, Single‐Arm, Phase II Trial of Sintilimab Plus Anlotinib for Metastatic Non‐Small Cell Lung Cancer After First‐Line PD‐(L)1 Inhibitor
Source: Cancer Med. 2025 Sep 4;14(17):e71191. doi: 10.1002/cam4.71191 (PMC12411266; doi:10.1002/cam4.71191)

**Supplementary Material**

**Figure S1.** Trial profile.


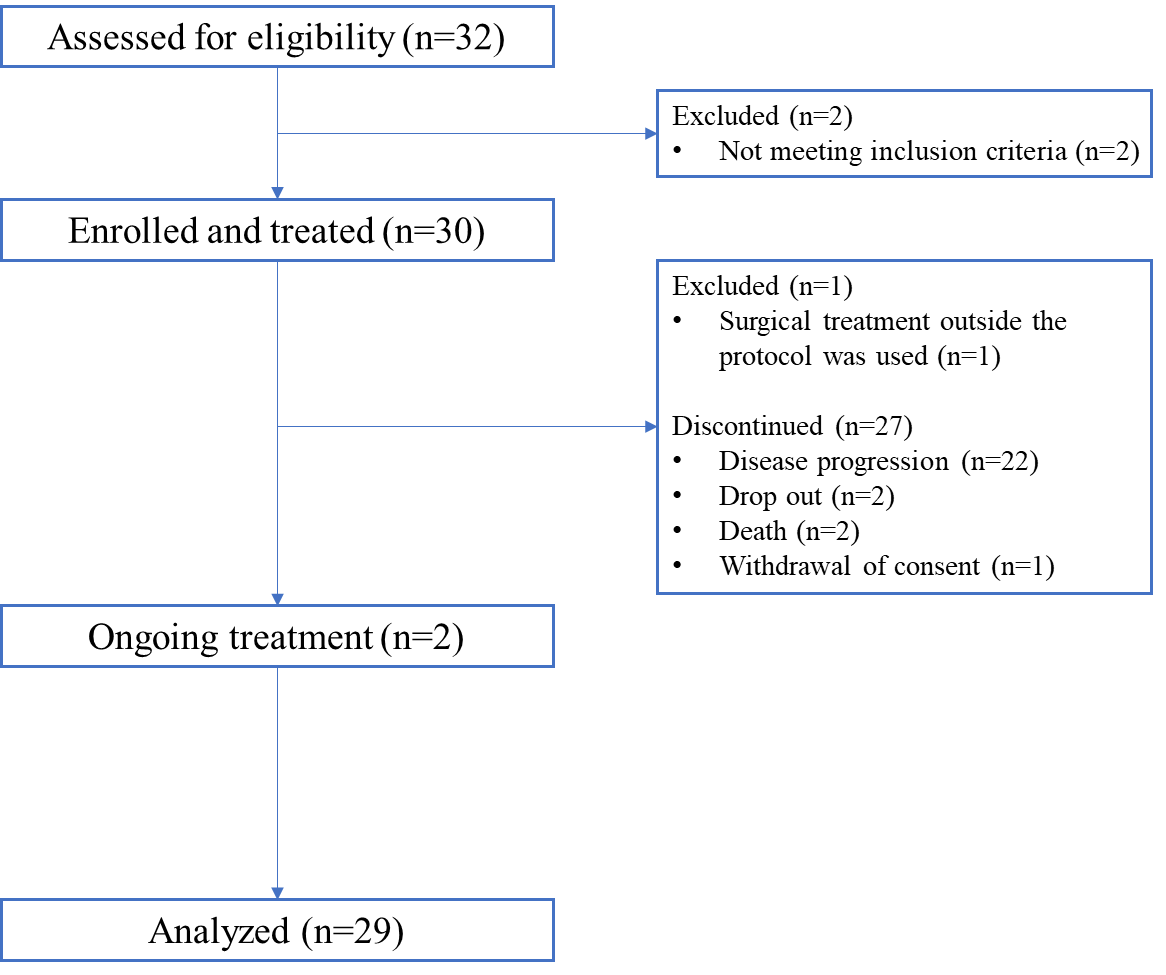

Supplement: Supplementary file 1 — Figure S1: cam471191‐sup‐0001‐Figure_S1.docx. [file CAM4-14-e71191-s001.docx]
